# Vestibular Evoked Myogenic Potential Produced by Bone-Conducted Stimuli: A Study on its Basics and Clinical Applications in Patients with Conductive and Sensorineural Hearing Loss and a Group with Vestibular Schawannoma

**Published:** 2013-06

**Authors:** Parvane Mahdi, Amin Amali, Akram Pourbakht, Alireza Karimi Yazdi, Ali Bassam

**Affiliations:** 1 *Department of Audiology, Faculty of Rehabilitation. Tehran University of Medical Sciences, Tehran, Iran.*; 2 *Department of Otorhinolaryngology-Head and Neck Surgery, Imam Khomeini Educational Complex Hospital, Tehran University of Medical Sciences, Tehran, Iran.*; 3 *Rehabilitation Research Center, Department of Audiology, Tehran University of Medical Sciences, Tehran, Iran.*

**Keywords:** Bone Conduction, Vestibular, VEMP

## Abstract

**Introduction:**

Vestibular evoked myogenic potential (VEMP) has recently been broadly studied in vestibular disorders. As it is evoked by loud sound stimulation, even mild conductive hearing loss may affect VEMP results. Bone-conducted (BC) stimulus is an alternative stimulation for evoking this response. This study aims to assess the characteristics of BC-VEMP in different groups of patients.

**Materials and Methods:**

We performed a cross sectional analysis on 20 healthy volunteers with normal pure-tone audiometry as a control group; and on a group of patients consisted of 20 participants with conductive hearing loss, five with bilateral sensorineural hearing loss and four with vestibular schawannoma. AC and BC-VEMP were performed in all participants.

**Results:**

In control group the VEMP responses to both kinds of stimuli had an acceptable morphology and consisted of p13 and n23 waves. Latency value of these main components in each type of stimulus was not significantly different (P>0.05). However, the mean amplitude was larger in BC modality than AC stimulation (P=0.025). In the group with conductive hearing loss, the VEMP response was absent in fifteen (46.87%) of the 32 ears using the AC method, whereas all (100%) displayed positive elicitability of VEMP by BC method. Normal VEMP responses in both stimuli were evoked in all patients with sensorineural hearing loss. In patients with unilateral vestibular schwannomas (VS), 2 (50.00%) had neither AC-VEMP nor BC-VEMP.

**Conclusion:**

Auditory stimuli delivered by bone conduction can evoke VEMP response. These responses are of vestibular origin and can be used in vestibular evaluation of patients with conductive hearing loss.

## Introduction

The Vestibular Evoked Myogenic Potentials (VEMPs) by definition are vestibulo-cervical reflexes elicited by intense sounds, upon the stimulation of saccule, and are collected from the tonically contracted sternocleidomastoid muscle (SCM) (1,2). As its simplicity and noninvasiveness in saccule and inferior vestibular nerve evaluation and the importance of considering each component of the vestibular apparatus to facilitate the complete assessment of patients with disequilibrium, the value of this test is self evidenced. So far, VEMP has considered to be a diagnostic tool for identifying many kinds of peripheral and central vestibular disorders (3,4). 

A great variety of recordings and stimulus parameters have been employed to obtain this response (5,6). Although air-conducted VEMP (AC-VEMP) can be recorded from patients with profound sensorineural hearing loss, the shortcoming to this method is its susceptibility to even mild conductive hearing loss. That is, as the stimulation threshold of the saccule is so high, the intensity near the maximum output of a device (i.e.,>90 dBnHL) is applied for its adequate actuation, so, the probability of detecting a VEMP via AC stimulus is dependent on the integrity of sound transmission through the middle ear conductive mechanism to the inner ear. When stimulating sound is attenuated by middle ear pathology, the amount of sound reaching saccule is reduced and VEMP is expected to be poorly elicited (2,7-11). To that end, an accurate vestibular assessment of patients with disequilibrium using AC-VEMP can be challenging when a conductive component of hearing loss is also coexisted. 

Bone-conducted (BC) stimulus is a recently described clinical method of stimulation for the needed recording of VEMP in patients with conductive hearing loss (7,8,12). This route of stimulation circumvents the middle ear conductive apparatus and results in a direct stimulation of vestibular end organ. 

Application of BC-VEMP in vestibular assessment of a variety of conductive patients have been reported; Sheykholeslami et al (2001), obtained BC-VEMP in patients with congenital atresia of the external auditory canal and reported short tone burst evoked potentials with better waveform morphology than clicks at the same subjective intensity in all recordings (13). 

Yang et al (2003), assessed the AC-VEMP elicitation in patients with chronic otitis media and reported the absence of response in 9(41%) of 22 affected ears and stated that when stimulating sound is attenuated by middle ear pathology, VEMPs are expected to be poorly elicited (14).

In a prospective study of Trivelli et al (2010), saccular evaluation of patients with otosclerosis were performed, using both AC and BC-VEMP and reported more response elicitability in BC compared to AC stimuli, 38.1% in contrast to 21.4% and stated larger ABG in those with absent response (15). As a new method for evoking vestibulocollic reflexes, it would be rational to identify its relative usefulness and characteristic in the field of vestibular function testing, if the procedure is to have generalized clinical application. Regarding the dearth of literature in this arena, we aimed at determining the properties of BC-VEMP responses compared to AC-VEMP in different groups of patients. In essence we want to elucidate whether BC stimulation provides the same information as AC stimulation. 

Such a study should give an insight in to the application of BC-VEMP in conductive patients, of whom recording of AC-VEMP is impossible for most of the time. 

## Materials and Methods

*Participants*

We performed a cross sectional analysis on a study sample consisted of 20 (9 male and 11 female) healthy volunteers with normal pure-tone audiometry and without any history of auditory and vestibular symptoms as a control group; and on a group of patients consisted of 20 (13 male/7 female) with conductive hearing loss and no vestibular complains, five (2 male/3 female) with bilateral sensorineural hearing loss and no vestibular complains and four (1 male/ 3 female) with vestibular schawannoma. 

*Study* *Protocol *

After an otoscopic examination and pure tone audiometry, all candidates subjected to VEMP testing using a clinical device EPIC-Plus (LABAT, Italy). 

Tone bursts of 500 Hz with a duration of 8 ms were administered at a rate of 5.1/sec. The stimuli intensities were 95dBnHL and 70dBnHL for AC and BC stimulation, respectively. The BC stimuli used to evoke the VEMP were delivered to the participants ear using clinical bone vibrator (B71, Radioear Corporation) placed on the mastoid process and the AC stimuli were applied using TDH39 headphone. The external auditory canals were plugged to minimize osseotympanic bone conduction. 

During the recording, participants remained in a sitting position, with a maximum head rotation to the contralateral side of the stimulated ear for constant and strong contraction of the SCM muscle. 

Muscle activation was monitored via a feedback method. The active electrode was placed over the middle portion of the ipsilateral SCM muscle body, the reference and ground electrodes were placed over the upper sternum and midline forehead, respectively. 

The recordings were performed in 100 ms windows. Myogenic potentials were averaged and bandpass filtered (10–2000 Hz) over a series of 150 stimuli. To ensure that the responses were originated from vestibular structures and were not an artifact, the responses were recorded twice in both sides to confirm the replication. 

The measured parameters were the peak latency (in ms) of the two early waves, p13 and n23, the peak to peak amplitude of the p13–n23 waves (in µv) and side to side differences in amplitudes were expressed as an amplitude ratio (AR), calculated using the following formula: AR= (Al-As) / (Al+As), where Al and As are the larger and smaller amplitudes, respectively, obtained from stimulating each ear. Direct comparison of the VEMP in response to AC and BC stimuli in the same participants, using the same stimulus, recording and monitoring methods was performed in all participants. 

The study protocol was approved by the Ethics Committee of Tehran University of Medical Sciences, while all participants were requested to fill an informed consent.

*Data Analysis*

SPSS software, version 11.5 (Chicago, IL, USA), was employed for statistical evaluation. Comparative analysis of the different parameters between AC and BC-VEMP findings was completed by student t-test. Correlations between different parameters were assessed using Pearson’s correlation test. In all statistical procedures, instances with a P<0.05 were considered as statistically significant.

## Results

*Response properties in normal volunteers*

Demographic features of all participants are shown in Table 1. In control group 40 ears (20 right, 20 left) were included. The VEMP responses to both kinds of stimuli had an acceptable morphology and consisted of p13 and n23 waves (Table. 1). 

The mean latencies of p13 and n23 of the BC-VEMP were 13.68 ±1.43 ms and 21.95 ± 3.70 ms, respectively. The corresponding values for AC-VEMP were 14.57±2.31 ms and 23.65±4.11 ms. Comparison of peak latency of these main components of VEMP for each type of stimulus was not significantly different (P>0.05). As it is shown in table 2, the mean peak to peak amplitude against EMG activity was larger in BC modality than that obtained from AC mode of stimulation, representing a significant difference (P=0.025). The mean AR of AC-VEMP (0.17±0.13) did not significantly differ from that obtained in BC-VEMP (0.24±0.21) (P=0.104) (Table. 2).

**Table 1:** Demographic Features of All Participants in the Study

Parameters	Control group		CHL* group	SNHL** group	VS*** group	
N		20 subjects,40 ears	20 subjects, 32 ears	5 subjects, 10 ears		4 subjects, 4 ears
Age		32.44±8.96	48.62±9.51	51.38±6.92		30.66±3.12
Gender (male/female)		9/11	13/7	2/3		1/3
Duration of disease		-----	4.63±4.10	12.86±7.11		5.12±4.64

*Conductive Hearing Loss, **Sensorineural Hearing Loss, *** Vestibular Schwannomas

**Table 2:** Comparison of VEMP Parameters Between AC and BC Stimuli

Parameter	AC stimuli	BC stimuli	P-value
**Latency**			
p13(ms)	14.57±2.31	13.68 ±1.43	0.154
n23(ms)	23.65±4.11	21.95 ± 3.70	0.094
			
**Amplitude**			
P13-n23(µv)	*75.45±21.17*	83.64±39.13	0.025*
AR	0.17±0.13	0.24±0.21	0.104

*Statistically significant

*Findings From a Group of Patients With Conductive Hearing Loss*

**Fig1:**
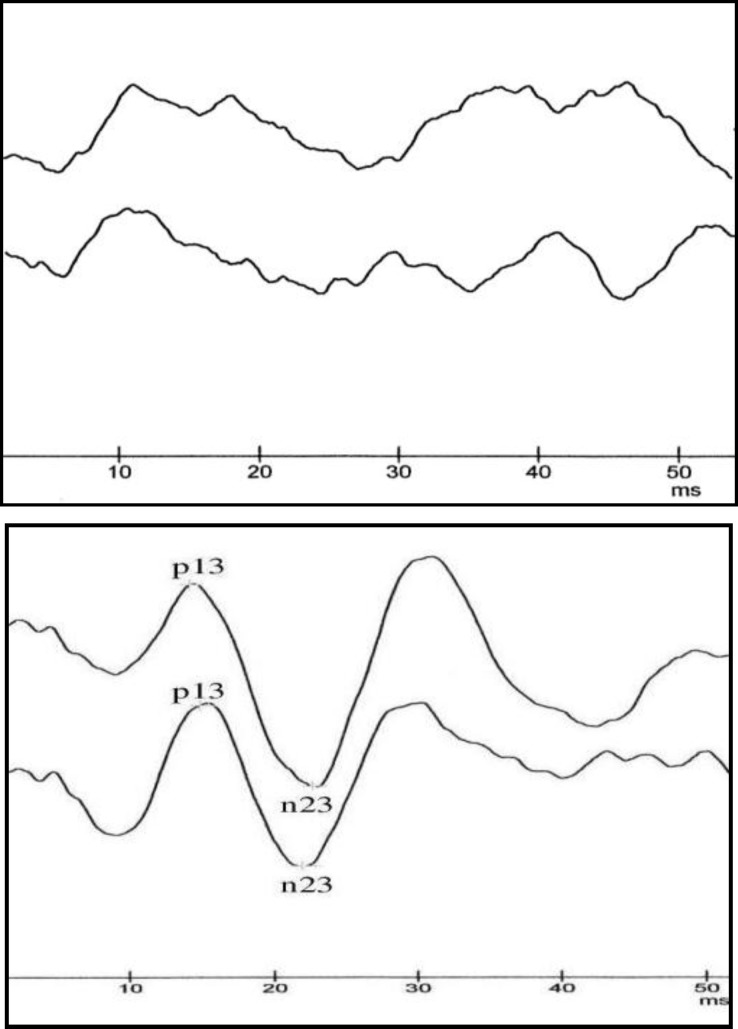
AC and BC-VEMP Recorded From the SCM of a 32 Year Old Patient With Severe Conductive Hearing Loss. In AC Stimulation (Left Figure) No Response was Detected in 2 Trials, However in BC Stimulation (Right Picture) Normal Responses Were Repeated in Both Trials

In the sample of the consecutively admitted 20 patients with conductive hearing loss, 32 ears (15 Right/ 17 Left) were included. The VEMP response was found to be absent in fifteen (46.87%) of the 32 ears using the AC method, whereas all (100%) displayed positive elicitability of VEMP by the BC method. In AC-VEMP nonresponsive ears, the median ABG in 500Hz was 32.3±17.8 dB in contrast to the gap of 21.5±14.9 dB in ears with recorded AC-VEMP. Figure 1 illustrates the potentials obtained from the ears with conductive hearing loss, in both AC and BC stimulation.

*Findings From Sensorineural Group*

Five patients with bilateral sensorineural hearing loss were included. Neither of these patients had the symptom nor the complaint of vestibular abnormalities. In the same vein to AC stimulus, short latency peaks in BC recordings of VEMP were preserved in all participants. 

*Findings From Vestibular Schwannomas Group*Four patients with definite diagnosis of unilateral vestibular schwannomas (VS) participated in this study. In two (50.00%) of four affected ears, AC-VEMP could not be detected, of which the BC-VEMP were also absent, too. 

## Discussion

Vestibular apparatus in amphibians and mammals can be activated by vibration (15,17), additionally, as reported by Young et al (1977), in squirrel monkey, primary afferents from all vestibular end organs were sensitive to acoustic as well as vibration sound (18). 

Vestibular symptoms such as illusion of self-motion and nystagmus following vibration of the head using 120-280 Hz stimuli were reported by Lackner and Graybiel (17,19). 

Recently, bone-conducted sound has been used in an attempt to evoke the VEMP from SCM. Specifically, Sheykholeslami et al (2000) elicited VEMP after presenting click stimuli through a bone conduction transducer at the tip of mastoid. They reported that the largest amplitude of BC-VEMP occurred in response to tone burst of low frequency (8). In a similar study, Welgampola and associates reported that response to bone conduction stimulation were often bilateral, with the largest VEMP recorded from the ipsilateral SCM. They reported that VEMP could be elicited at lower sound levels for stimuli delivered by bone conduction (7). 

The present study was one of the few studies to evaluate the properties of BC-VEMP in different groups of patients. We found that BC sound of 500Hz with 70 dBnHL produced typical VEMP response in all 20 normal participants. The latency of p13 and n23 potentials were almost comparable between AC and BC modality of stimulation, the reported larger amplitude in BC mode could be attributed to the more effective stimulation of saccule by means of bone vibrator, which is in agreement with previous studies findings (7,8). 

Our patients in CHL group, showed lower response rates in AC-VEMP than BC-VEMP, although the correlation between the tendency of AC-VEMP elicitability with the ABG was statistically significant (P= 0.002), and it was in accordance with the findings of Halmagy et al (1995), who reported the absence of AC-VEMP in patients with an ABG of more than 20 dB (11). 

BC-VEMP response was remained intact in our patients with sensorineural hearing loss, as has been noted regarding the AC-VEMP, indicating the vestibular origin of both AC and BC-VEMP and their independency to cochlear integrity. Last, the similarity in AC and BC-VEMP detectability was witnessed in patients with VS. The absence of both AC and BC stimulation on the side of the lesion in two patients, suggesting that tumors were proved to originate from the inferior division of vestibular nerve and also leaded to the findings that the same diagnostic information was provided in both AC and BC stimulation. Accordingly, Miyamoto et al (2006), compared AC and BC-VEMP in normal volunteers and patients with unilateral vestibular disorder and concluded that the results of BC-VEMP were the same as those for AC-VEMP, at least, for patients without conductive hearing loss (20). 

## Conclusion

Taken together, from the results of the present study along with previous investigations, it can be concluded that with the application of auditory stimulation as a bone conduction, VEMP can be recorded and the BC-VEMP had an adequate morphology and amplitude and it was of vestibular origin, thereby enabling the vestibular evaluation of patients with conductive hearing loss. We recommend further studies, with larger sample size and using different kinds of patients for more analysis of BC-VEMP responses characteristics.
